# Long-Term Survival after Curative Resection for Postoperative Dissemination of Pancreatic Ductal Adenocarcinoma: A Case Report

**DOI:** 10.70352/scrj.cr.24-0022

**Published:** 2025-04-02

**Authors:** Yoshitaka Shimamaki, Makoto Takahashi, Taku Higashihara, Tatsuya Hayashi, Yasuhiro Morita, Takeshi Azuma, Dai Inoue, Haruka Okada, Masayuki Ohtsuka

**Affiliations:** 1 Department of General Surgery, Tokyo Metropolitan Tama Medical Center, Fuchu, Tokyo, Japan; 2 Division of Urology, Tokyo Metropolitan Tama Medical Center, Fuchu, Tokyo, Japan; 3 Department of Gastroenterology and Hepatology, Tokyo Metropolitan Tama Medical Center, Fuchu, Tokyo, Japan; 4 Department of Clinical Genomics, Tokyo Metropolitan Tama Medical Center, Fuchu, Tokyo, Japan; 5 Department of Pathology, Tokyo Metropolitan Tama Medical Center, Fuchu, Tokyo, Japan; 6 Department of General Surgery, Graduate School of Medicine, Chiba University, Chiba, Chiba, Japan

**Keywords:** pancreatic ductal adenocarcinoma, dissemination, surgery, chemotherapy, long-term survival

## Abstract

**INTRODUCTION:**

Pancreatic ductal adenocarcinoma (PDAC) has a very poor prognosis and high mortality. The prognosis for recurrence after surgery is extremely poor. Resection for disseminations of PDAC is not recommended.

**CASE PRESENTATION:**

The patient was a 69-year-old woman with a pancreatic tumor that was detected with computed tomography (CT) during a postoperative colon cancer checkup. She was suspected of having pancreatic body cancer without distant metastasis. Distal pancreatectomy with celiac axis resection was performed. Postoperative pathological examination revealed an invasive ductal adenocarcinoma with lymph node metastasis (pT4N1M0, stage III). Postoperatively, she received adjuvant chemotherapy containing gemcitabine and S-1 for 1 year and 4 months, and S-1 monotherapy for 1 year. Six years and 2 months after the initial surgery, her serum carbohydrate antigen 19-9 level elevated, and CT revealed soft tissue in front of the left kidney. Positron emission tomography/CT also revealed high fluorine-18 fluorodeoxyglucose uptake in the tissue. Accordingly, the patient was diagnosed with dissemination of PDAC. The patient was administered chemotherapy with gemcitabine and S-1. One year and 6 months after the diagnosis of dissemination, CT revealed reduction of the nodule. Therefore, we decided to eliminate this dissemination. A left nephrectomy and partial gastrectomy were performed. Histopathological examination confirmed dissemination of PDAC. The patient refused adjuvant chemotherapy. No evidence of recurrence has been observed for 13 years and 3 months since the initial surgery, and 5 years and 1 month since the resection of the dissemination.

**CONCLUSIONS:**

This case showed a recurrence of dissemination after radical PDAC surgery, and the patient showed long-term survival without recurrence after dissemination resection. Resection of dissemination may confer long-term survival in selected patients.

## Abbreviations

CA
: celiac artery
CA19-9
: carbohydrate antigen 19-9
CH
: common hepatic artery
CT
: computed tomography
DPCAR
: distal pancreatectomy with celiac axis resection
EUS-FNA
: endoscopic ultrasound-guided fine needle aspiration
FDG
: fluorodeoxyglucose
FOLFIRINOX
: oxaliplatin, leucovorin, irinotecan, plus 5-fluorouracil
GEM
: gemcitabine
GnP
: gemcitabine plus nanoparticle albumin bound paclitaxel
GS
: gemcitabine plus S-1 combination therapy
LGA
: left gastric artery
MDT
: multidisciplinary team
MST
: median survival time
PDAC
: pancreatic ductal adenocarcinoma
PET
: positron emission tomography
RECIST
: Response Evaluation Criteria in Solid Tumors
SD
: stable disease
SUVmax
: maximum standardized uptake value
UICC
: Union for International Cancer Control

## INTRODUCTION

Pancreatic ductal adenocarcinoma (PDAC) has a very poor prognosis and high mortality rate. Despite recent progress in diagnostic imaging modalities, surgical procedures, radiotherapy, and chemotherapy, prognosis remains poor.^1)^ Patients with metastatic PDAC have a 5-year overall survival of only 2%.^2)^ Distant metastases of PDAC often occur in the liver, lungs, lymph nodes, and peritoneum. Successful surgical intervention in patients with an isolated lung recurrence and recurrence in the remnant pancreas has been reported.^3,4)^ However, cases in which PDAC dissemination is surgically resected are extremely rare. This is an extremely rare case of PDAC dissemination after radial curation in a patient who survived without recurrence for 5 years and 1 month after dissemination resection.

## CASE PRESENTATION

**Fig. 1:**
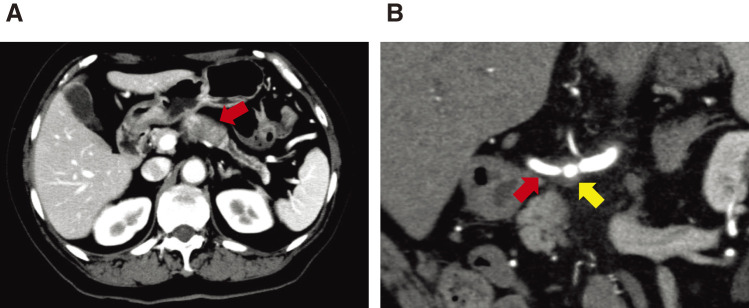
Radiological examinations before initial surgery. (**A**) Computed tomography (CT) showing a 43-mm hypovascular tumor in the pancreatic body (arrow). (**B**) The tumor contacts with the common hepatic artery (<180°) (red arrow), and the celiac artery (<180°) (yellow arrow).

**Fig. 2:**
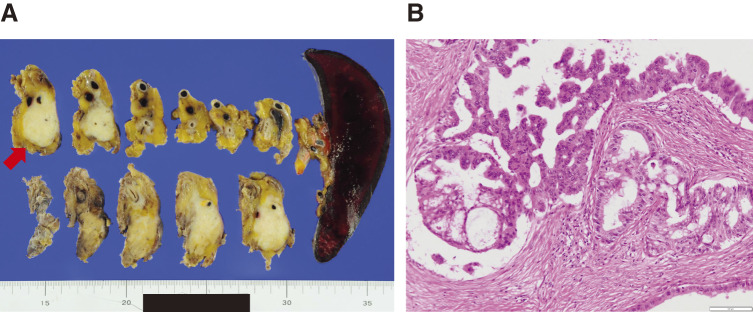
Pathological findings in the first surgery. (**A**) Macroscopic examination of the resected specimen identified a 50-mm nodule in the pancreatic body (arrow). (**B**) Histopathological findings (hematoxylin-eosin staining) showing invasive ductal adenocarcinoma with lymphatic invasion. Scale bar: 100 µm.

A 69-year-old woman was referred to our department in March 2011 after a follow-up computed tomography (CT) performed after sigmoid colon cancer surgery showed a pancreatic tumor. Abdominal CT revealed a 43-mm hypovascular tumor in contact with the common hepatic artery (CHA) (<180°) and celiac artery (CA) (<180°) in the pancreatic body (**Figs. 1A**, **1B**). The initial laboratory findings were unremarkable except for a high level of carbohydrate antigen 19-9 (CA19-9; 60.3 U/ml). No metastasis was observed. Imaging clearly indicated pancreatic cancer; however, we were unable to perform endoscopic ultrasound-guided fine needle aspiration (EUS-FNA) in our facility. Therefore, a preoperative histopathological diagnosis was not performed. A distal pancreatectomy with celiac axis resection (DPCAR) was planned. Coil embolization of the CHA and left gastric artery (LGA) was performed 4 days before surgery to prevent ischemic changes in the liver and stomach, as was common practice at that time.^5,6)^ The histopathological diagnosis following DPCAR with clear margins confirmed invasive ductal adenocarcinoma, pT4 (50 mm × 45 mm), N1, M0, pStage III, according to the Union for International Cancer Control (UICC) 8th edition, as well as according to the Japanese Classification of Pancreatic Carcinoma, 8th edition, by the Japan Pancreas Society^7)^ (**Figs. 2A**, **2B**). She experienced a postoperative pancreatic fistula (Clavien-Dindo grade IIIa),^8)^ which was treated with drainage and antibiotic agents and was discharged on postoperative day 36.

As postoperative adjuvant chemotherapy, gemcitabine (GEM) plus S-1 combination therapy (GS) was administered for 1 year and 4 months, while S-1 monotherapy was administered for 1 year. GEM was administered at a dose of 1000 mg/m^2^ on days 1 and 8 of a 21-day cycle, and S-1 was administered at a dose of 40 mg/m^2^ twice daily on days 1–14 followed by a 7-day rest period. Thereafter, the CA19-9 level was within the normal range, and follow-up CT revealed no local recurrence.

**Fig. 3:**
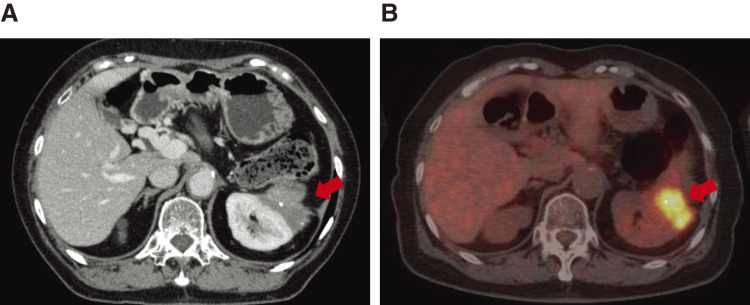
Radiological examinations of dissemination. (**A**) CT showing soft tissue in front of left kidney (arrow). (**B**) Positron emission tomography (PET)/CT shows fluorodeoxyglucose (FDG) uptake in the tissue (arrow).

**Fig. 4:**
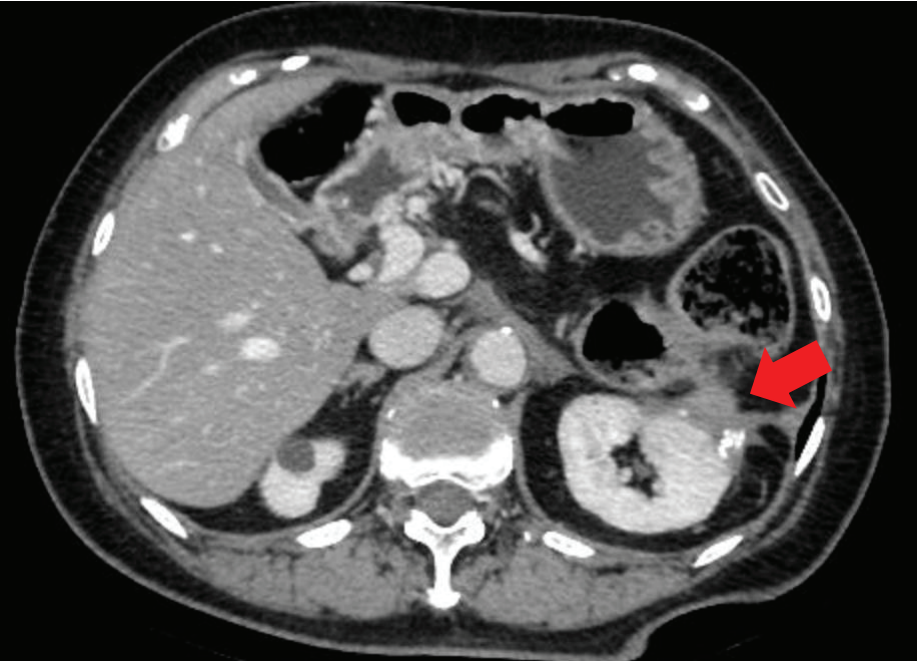
Radiological findings after chemotherapy. The size of dissemination was slightly reduced (arrow).

**Fig. 5:**
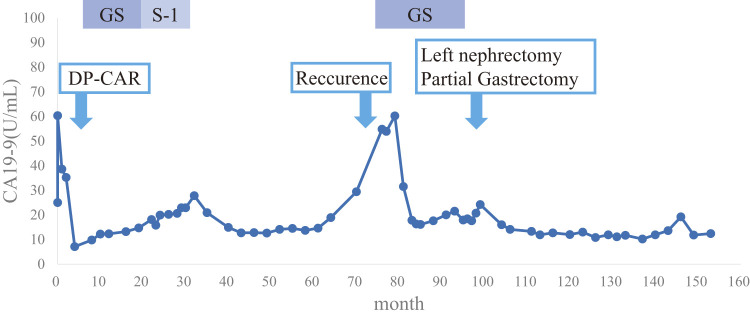
Serum carbohydrate antigen 19–9 (CA19-9) levels. The CA19-9 level was elevated at the time of recurrence but normalized with chemotherapy and a second surgery.

**Fig. 6:**
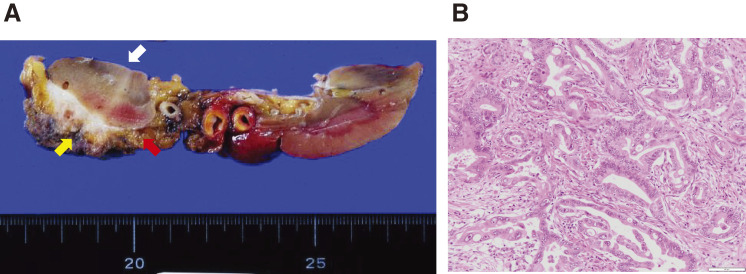
Pathological findings of second surgery. (**A**) Macroscopic examination of the resected specimen identified tissue (red arrow) between stomach (yellow arrow) and left kidney (white arrow). (**B**) Histopathological findings (hematoxylin-eosin staining) showing adenocarcinoma compatible with metastasis of pancreatic ductal adenocarcinoma. Scale bar: 100 µm.

**Fig. 7:**
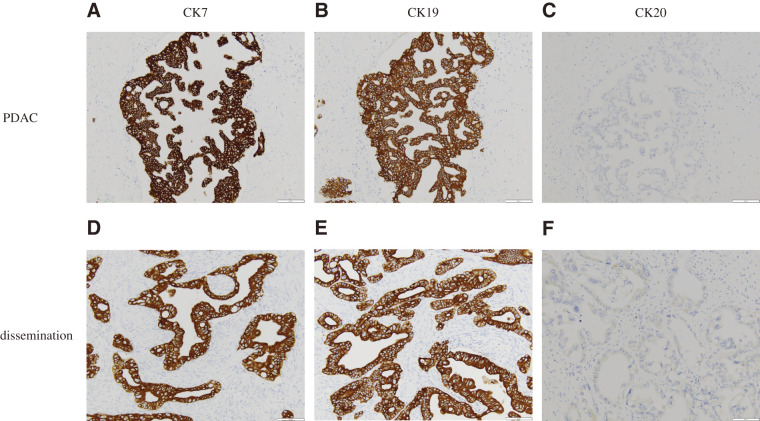
Immunostaining findings. The staining patterns of pancreatic ductal adenocarcinoma (PDAC) and dissemination were similar. (**A**) CK7 was positive in the PDAC. (**B**) CK19 was positive in the PDAC. (**C**) CK20 was negative in the PDAC. (**D**) CK7 was positive in the dissemination. (**E**) CK19 was positive in the dissemination. (**F**) CK20 was negative in the dissemination.

In June 2017, 6 years and 2 months after the initial surgery, the CA19-9 level was elevated (54.8 U/mL), and abdominal enhanced CT revealed soft tissue in front of the left kidney (**Fig. 3A**). Positron emission tomography (PET)/CT showed fluorodeoxyglucose (FDG) uptake in the tissue with a maximum standardized uptake value (SUVmax) of 13.3 (**Fig. 3B**). EUS showed soft tissue, which was diagnosed as metastatic adenocarcinoma using FNA cytology. Accordingly, the patient was diagnosed with disseminated PDAC. GS therapy was administered for 1 year and 6 months. Enhanced CT showed a reduction of the soft tissue (**Fig. 4**), indicating stable disease (SD) according to the Response Evaluation Criteria in Solid Tumors (RECIST). The CA19-9 level decreased to within the normal range (**Fig. 5**). Because the dissemination was isolated, the size was slightly reduced, and no new lesions appeared, she opted for removal at a multidisciplinary team (MDT) meeting. In June 2019, 8 years and 2 months after the initial surgery, left nephrectomy and partial gastrectomy were performed because of a suspected stomach invasion. Macroscopic examination of the resected specimen revealed soft tissue extending from the renal cortex to the gastric serosa (**Fig. 6A**). Pathologically, the disseminated nodules showed a morphology similar to that of the initial surgical specimen after hematoxylin and eosin staining (**Fig. 6B**). In addition, immunostaining showed similar staining morphology, with CK7 and CK19 positivity and CK20 negativity in both specimens (**Fig. 7**). Based on these findings, the patient was diagnosed with PDAC dissemination. The patient’s postoperative course was uneventful, and she was discharged on postoperative day 19. The patient refused to undergo adjuvant chemotherapy. She remained alive for 5 years and 1 month after the second surgery and 13 years and 3 months after the first surgery, without recurrence.

## DISCUSSION

The prognosis of postoperative recurrence of PDAC is extremely poor.^1,9)^ PDAC generally progresses rapidly. According to recent clinical trials on postoperative therapy after radical resection, the 5-year survival rate was 44.1% in the S-1 group and 24.4% in the GEM group, indicating poor prognosis compared with other cancers.^10)^ One reason for this is that recurrence is almost inevitable even after radical resection of PDAC, with a reported 5-year recurrence-free survival rate of 3%.^11)^

Recently, neoadjuvant chemotherapy has been recommended for managing PDAC, even with resectable tumors, which contributes to improved prognosis.^12)^ However, in 2011, evidence supporting this approach remained insufficient. Therefore, in this case, surgery was performed in the absence of neoadjuvant chemotherapy.

The administration of S-1 monotherapy as adjuvant chemotherapy after radical resection of PDAC is now a common practice.^10)^ However, in 2011, GS therapy was administered to the patient,^13,14)^ based on the findings of several reports that demonstrated the efficacy of its use.

Recently, two regimens, oxaliplatin, leucovorin, irinotecan, plus 5-fluorouracil (FOLFIRINOX) and GEM plus nanoparticle albumin-bound paclitaxel (GnP), have been used as standard treatments for metastatic PDAC. The median survival time (MST) was reported to be 11.1 months for FOLFIRINOX^15)^ and 8.5 months for GnP.^16)^ After the recurrence of PDAC, FOLFIRINOX or GnP therapy were the preferred treatment options for this case as well. However, following consultation with the patient, GS therapy was re-administered after recurrence due to its high tolerability.

The most common initial sites of recurrence for PDAC were local recurrence (71.8%) and liver metastasis (61.5%), followed by peritoneal recurrence (10%).^11)^ In this case, the mechanism by which PDAC initially recurred with dissemination is unclear; however, potential pathways include lymphatic invasion from lymph node metastasis and the possibility of intraoperative seeding. We had not performed EUS-FNA before the first surgery; therefore, the cause of dissemination was not needle-tract seeding.

Systemic chemotherapy is the standard treatment for metastatic PDAC recurrence. Recent studies have suggested the use of resection for metastases in selected patients. The Japanese Clinical Practice Guidelines for Pancreatic Cancer 2022 proposed the resection of remnant pancreatic recurrence and lung metastasis after carefully assessing the indications.^17)^ It has been proposed that resection of other metastatic sites should not be performed. Some researchers insist that the resection of liver metastases is beneficial. There are reports that in cases of resection of recurrent liver metastasis, good prognosis has been achieved in patients who maintained a solitary metastasis with long-term chemotherapy and had a long time before recurrence.^18–20)^ Frigerio et al. resected PDAC liver metastases after chemotherapy in 25 of 535 patients, with an overall survival of 56 months and a disease-free survival of 27 months.^21)^ Saito et al. defined patients with oligo-like liver metastasis as those with all of the following three conditions: long recurrence-free interval (≥6 months), long stable disease interval (>3 months), and three or fewer recurrent tumors. Patients with oligo-like liver metastasis had a significantly better prognosis for overall survival after recurrence compared with that of patients without oligo-like liver metastasis.^22)^ Kleeff et al. reported that patients with a prolonged interval (>9 months) between resection and recurrence were more likely to benefit from resection than those with recurrence within 9 months (MST 17.0 vs. 7.4 months; P = 0.004).^23)^ Therefore, for patients with liver metastases of PDAC, the number of lesions and time to recurrence may be useful criteria for resection. Although PDAC is generally considered to progress rapidly, there is a subset with slower progression. Within this group, it has been suggested that there may be a population for whom surgical resection of postoperative recurrent liver metastases can be considered.

There have been a few reports of resection of so-called oligometastasis. We also reported a case of resection of gastric and gallbladder metastases after PDAC surgery.^24)^ However, a case of resection for postoperative dissemination is extremely rare. A literature search revealed that only 3 patients underwent postoperative peritoneal resection, including the present case (**Table 1**).^25,26)^ Almost all the patients exhibited a relatively long time to relapse and maintained a solitary metastasis. These results suggest that a longer time to relapse may be associated with longer survival. Further studies are required to determine the indications for the resection of dissemination.

**Table 1:** Details of previous reports

No.	Age (y), Sex	pTNM	Operation	Adjuvant chemo-therapy	Time to recurrence	Chemo-therapy	Reoperation	Survival time
1^25)^	63, Male	T3N2M0	SSPPD	GEM	17 months	S-1	Right hemicolectomy Right partial nephrectomy	36 months
2^26)^	73, Female	T3N0M0	DP	GEM	15 months	None	Partial small intestine resection	11 months
Present case	79, Female	T4aN1M0	DPCAR	GS, S-1	74 months	GS	Left nephrectomy Partial gastrectomy	61 months

SSPPD, subtotal stomach preserved pancreaticoduodenectomy; DP, distal pancreatectomy; DPCAR, distal pancreatectomy with celiac axis resection; GEM, gemcitabine; GS, gemcitabine plus S-1 combination therapy

This case involved the disseminated recurrence of PDAC. Single recurrence, extended interval before recurrence, good response to chemotherapy, and a decrease in tumor markers can all contribute to long-term recurrence-free survival after resection. PET/CT evaluation was not performed immediately before resection in this case; therefore, valuable information supporting the decisions made was unfortunately unavailable.^9,27)^

## CONCLUSION

This study is about an extremely rare case of PDAC dissemination after radical surgery in a patient who survived without recurrence for 13 years and 3 months after DPCAR, and 5 years and 1 month after nephrectomy and partial gastrectomy. Our findings suggest that the resection of dissemination may confer long-term survival in selected patients. Time to postoperative recurrence may be a useful criterion for determining whether radical resection should be performed.

## DECLARATIONS

### Funding

The authors declare that they have no conflicts of interest.

### Authors’ contributions

YS and MT gathered patient data and wrote the manuscript. YM participated in both surgical procedures.

DI performed EUS-FNA before the second surgery.

TA performed the second surgery.

HO performed the pathological diagnosis.

MT, THa, THi, YM, and MO discussed the data with YS and helped write the manuscript.

All the authors approved the final manuscript.

### Availability of data and materials

Data sharing is not applicable to this article.

### Ethics approval and consent to participate

Ethics approval is not applicable because this is a case report.

### Consent for publication

Written informed consent was obtained from the patient for the publication of this case report and the accompanying images.

### Competing interests

The authors declare that they have no competing interests.
